# Role of Chemosensory TRP Channels in Lung Cancer

**DOI:** 10.3390/ph11040090

**Published:** 2018-09-21

**Authors:** Thomas R. H. Büch, Eva A. M. Büch, Ingrid Boekhoff, Dirk Steinritz, Achim Aigner

**Affiliations:** 1 Rudolf Boehm-Institute for Pharmacology and Toxicology, Clinical Pharmacology, Leipzig University, Haertelstrasse 16-18, D-04107 Leipzig, Germany; eva.schaefer@lrz.uni-muenchen.de; 2 Walther Straub Institute of Pharmacology and Toxicology, Ludwig-Maximilian University, D-80336 Munich, Germany; ingrid.boekhoff@lrz.uni-muenchen.de (I.B.); dirk.steinritz@lrz.uni-muenchen.de (D.S.); 3 Bundeswehr Institute of Pharmacology and Toxicology, Neuherbergstr. 11, D-80937 Munich, Germany

**Keywords:** TRP, TRPA1, lung cancer, TRPV1, tumor-promoting effects

## Abstract

Transient receptor potential (TRP) channels represent a large family of cation channels and many members of the TRP family have been shown to act as polymodal receptor molecules for irritative or potentially harmful substances. These chemosensory TRP channels have been extensively characterized in primary sensory and neuronal cells. However, in recent years the functional expression of these proteins in non-neuronal cells, e.g., in the epithelial lining of the respiratory tract has been confirmed. Notably, these proteins have also been described in a number of cancer types. As sensor molecules for noxious compounds, chemosensory TRP channels are involved in cell defense mechanisms and influence cell survival following exposure to toxic substances via the modulation of apoptotic signaling. Of note, a number of cytostatic drugs or drug metabolites can activate these TRP channels, which could affect the therapeutic efficacy of these cytostatics. Moreover, toxic inhalational substances with potential involvement in lung carcinogenesis are well established TRP activators. In this review, we present a synopsis of data on the expression of chemosensory TRP channels in lung cancer cells and describe TRP agonists and TRP-dependent signaling pathways with potential relevance to tumor biology. Furthermore, we discuss a possible role of TRP channels in the non-genomic, tumor-promoting effects of inhalational carcinogens such as cigarette smoke.

## 1. Introduction

Lung cancer can be divided into four subtypes: adenocarcinoma (~40% of all cases), squamous carcinoma (30%), large cell carcinoma and small cell carcinoma (SCLC) (15% each) [1]. Due to their clinical similarities, adeno-, large cell and squamous cell carcinoma are often grouped as non-small cell lung carcinoma (NSCLC). In NSCLC therapy, remarkable progress has been made in the last ~15 years, including targeted therapies and immunotherapies such as EGFR blockers, dual kinase inhibitors or check point inhibitors [2,3,4] that are usually combined with classical and well-established chemotherapies (etoposide, cisplatin). However, there is still an urgent need for improved therapeutic strategies based on the interference of critical oncogenic signaling pathways. In the case of SCLC, the therapeutic options have even more limitations. SCLC patients often present with advanced disease at the time of diagnosis, i.e., with metastases, thus limiting local therapy options (surgery or other local intervention, local radiation) in favor of combination chemotherapy [5,6]. Due to its rapid proliferation rate, the initial response is often remarkably high, with, unfortunately, subsequent development of chemoresistance and disease progress, which highlights the need for novel target molecules and pathways and more detailed knowledge regarding underlying drivers of SCLC carcinogenesis [7].

To date, novel therapeutic approaches targeting specific oncogenic signaling pathways have focused on the interference of critical membrane receptors (e.g., receptor tyrosine kinases) or downstream signaling molecules (e.g., receptor-regulated kinases or transcription factors). However, the modulation of tumor-relevant ion channels (by stimulating tumor-inhibiting channels or by blocking tumor-promoting channels) represents an interesting alternative concept. Thus, TRP (transient receptor potential) channels are promising candidates for innovative anticancer therapies [8].

The discovery of TRP channels is related to a Drosophila mutant already described in the 1960s [9] showing an altered light reaction. The *TRP*-gene encoding a rhodopsin-activated calcium channel was cloned 20 years later [10]. Subsequent studies indicated the existence of multiple different TRP homologues in other species [11]. Thus, TRP channels comprise a large and divergent family of channel proteins, expressed in various tissues and cell types in vertebrates as well as in invertebrates. A common feature of the TRP superfamily of cation channels are six transmembrane segments and a certain sequence homology. On the other hand, the major differences to other families of ion channels lie in their diversity of cation selectivities and specific mechanisms of activation. Notably, a given TRP channel can be activated, and its response can be modulated by entirely different mechanisms, which has led to the concept of TRP channels as ‘multiple signal integrators’. Furthermore, another common feature seems to lie in the TRP channels’ response to all large classes of external stimuli such as light, sound, chemicals, temperature, changes in osmolarity and direct contact [12,13,14].

The TRP superfamily can be subdivided into six subfamilies that are ordered into two groups: group 1 comprises the subfamilies **TRPC** (canonical), **TRPV** (vanilloid), **TRPM** (melastatin) and **TRPA** (ankyrin), while group 2 consists of only two subfamilies, **TRPP** (polycystin) and **TRPML** (mucolipin) [12,15]. It is not possible to predict the mechanism(s) of activation of a given channel based on its affiliation to a subfamily. Moreover, natural compounds such as capsaicin or menthol activate the heat-sensitive TRPV1 and the cold-sensitive TRPM8 channels, defining these channels as not only temperature sensors but also chemosensory channels.

In the following, TRP subfamilies, which include chemosensory channels, e.g., TRPV, TRPM, and TRPA are briefly characterized. The ‘classical’ TRPC channels (for a review see: [16,17,18] are not chemosensory channels *sensu stricto* and are therefore omitted.

**TRPV** channels share about 25% sequence homology with the TRPC channels in a region spanning the transmembrane domains 5 and 6. TRPV1 is activated by heat (≥43 °C) [19] and chemicals such as endocannabinoids, anandamide, camphor and others, with low pH, ethanol, nicotine or pro-inflammatory cytokines leading to further enhancement of cation flux. Again, this leads to the concept of TRPV1 acting as a multiple signal integrator. TRPV2, TRPV3 and TRPV4, but not TRPV5 and V6, are also heat activatable [14].

The **TRPM** channels share about 20% amino acid sequence identity with the TRPC channels over the five C-terminal transmembrane domains and contain a TRP domain C-terminally to the transmembrane segments [20]. While the total length and sequence of their C-terminal regions shows major differences, they can be subdivided into three subfamilies: TRPM1/3, TRPM4/5 and TRPM6/7. Despite some similarities, TRPM2 and M8 do not form another subfamily. TRPM1 was the first TRPM identified in mammals [21], and in some melanoma cell lines its expression level inversely correlates with their metastatic potential. TRPM4 and M5 are voltage gated, calcium-activated, monovalent cation-selective channels (VCAMs), based on a short acidic stretch of six amino acids in the pore loops. TRPM2, M6 and M7 are characterized by a C-terminal kinase domain allowing for channel-independent signaling and are therefore designated as chanzymes. TRPM7 is a divalent-permeable cation channel that conducts, inter alia, Mg^2+^ ions, which in turn regulate the channel activity (as free Mg^2+^ or Mg-complexed nucleotides).

**TRPA1** is the only member of the TRPA channels to have been characterized in man so far (for a review see: [22]). It is characterized by the presence of around 17 ankyrin-repeats in its N-terminus. Furthermore, it contains a zinc binding site in the C-terminus and a calcium binding site in the N-terminus. To date, three cysteine residues have been identified that are thought to be responsible for TRPA1 activation through covalent modification [23,24]. TRPA1 is activated by a wide variety of substances [25]. Of note, beyond the ‘classical’ mechanism that relies on the binding of agents in their binding pocket (key lock principle), many electrophilic compounds activate TRPA1 through a specific mechanism, i.e., their covalent coupling to TRPA1. Below, activators of TRPA1, especially with tumor-biological relevance, will be discussed in more detail.

## 2. Airway Expression of Chemosensory TRP Channels

TRP channels are expressed throughout the airways from the nasal mucosa to the alveolo-capillary system. They have been found in neuronal cells in the airways, especially in the nerve endings of C fibers, but also in non-neuronal cells, e.g., in the pulmonary epithelium, in smooth muscle cells of the bronchi and vasculature as well as in pulmonary endothelial cells.

Regarding the function of TRP channels (in particular TRPA1 and TRPV1) in neuronal cells, a critical role as toxicant sensor has been established (for review: [26,27]). The activation of these sensory TRP channels in the nerve endings of the airways leads to the stimulation of protective reflexes (cough, increased mucus production, enhanced mucociliary clearance) [27,28,29,30], but also to neurogenic inflammation, which suggests an involvement of these channels in chronic obstructive lung diseases (asthma, COPD).

Of note, sensor TRPs, like TRPV4 or TRPA1 have recently been described in non-neuronal cells as well [31,32,33].

In fact, TRPV4 has been found expressed in many ciliary cells, e.g., in the ovarian duct [34], in cholangiocytes [35], and in bronchial epithelial cells [36,37]. The stimulation of tracheal cells with ATP, a well-known activator of ciliary beat frequency in the bronchial system, led to a receptor-operated calcium signal strongly dependent on TRPV4 [36]. Intriguingly, the activation of TRPV4 has been described as stimulating the release of ATP by airway epithelial cells, a process that is induced by cell swelling in a pathway involving pannexin 1, RhoA and myosin light chain phosphorylation [37]. These findings suggest that TRPV4 and ATP may form a part of a self-amplifying system with TRPV4 being responsible for ATP-promoted calcium signaling and the subsequent triggering of ATP release with activation.

The TRPA1 channel has long been regarded as a sensor protein for harmful stimuli (for a review, see: [25]) mainly expressed in neuronal and neuroendocrine cells. However, non-neuronal functions of this channel, especially in the context of the airway epithelium have been proposed in recent years (for a review, see: [26,38]).

## 3. TRP Channels and Cancer

The involvement of TRP channels in tumor-relevant processes is plausible since calcium *per se* as well as calcium-dependent signaling molecules, play a pivotal role in the regulation of proliferation, apoptosis and cellular differentiation [39,40,41,42]. In fact, an association with cancer has been suggested for a number of chemosensory TRP channels based on altered expression levels (up- or downregulation in cancerous tissue as compared to normal tissue) or functional studies (TRP-promoted stimulation of oncogenic signaling and/or tumor-promoting or inhibitory effects). Of note, most analyses of TRP channels in cancer cells did not find critical mutations in these proteins that would affect the channel activity, but instead altered the expression levels of wild-type channels on the mRNA and/or protein level [42].

It is remarkable that some members of the TRP family have been found to be involved in tumor-promoting processes, whereas other TRP channels have been linked with the suppression of tumor growth. The postulated opposing functions of TRP channels are in line with the complex role of calcium in the orchestration of cell growth as well as apoptosis. 

Regarding the family of **TRPM (melastatin)** channels, a number of members have been associated with tumor progression (see [43] for a review). For example, the founding member, TRPM1 was first described as a gene downregulated in melanoma cells as compared to benign melanocytes [21,44]. This led to the hypothesis of TRPM1 acting as a tumor suppressor (hence the name melastatin). In line with this assumption, the induction of cellular differentiation in melanoma cells by treatment with hexamethylenbisacetamide caused an upregulation of TRPM1 transcripts [45]. In contrast to the potential tumor suppressor TRPM1, other members of this family have been implicated in oncogenic processes. For example, TRPM8 (initially named Trp-p8) was first been described in a screening experiment analyzing upregulated transcripts in prostate cancer tissue [46]. Since then, a number of reports have shown that this channel is involved in various tumor-biology relevant processes in prostate cancer (for a review, see [47]).

Likewise, a role in cancer cells has been described for several members of the **TRPV** family. For example, an implication of TRPV1 in the regulation of apoptotic pathways induced by cannabinoids in gynecologic carcinoma was described [48,49,50]. Another member of the TRPV family, TRPV6, is overexpressed in prostate cancer [51] and the expression level has been correlated with tumor grading, suggestive of a potential oncogenic role of TRPV6 in this tumor entity. 

With respect to the **TRPA1** channel, most publications addressing a protective role in cancer cells focus on lung cancer (see below). Of note, the very first papers identifying and describing TRPA1 [52,53] associated this protein with a tumor-suppressor function, since its expression was downregulated in tumor cells. It was only later that studies identified this novel protein as an important chemosensor for pain-eliciting substances or potentially harmful irritants [54]. While this finding shifted the interest in the TRPA1 field from tumor cells to neuronal or toxicological aspects of this channel, it was also found that TRPA1 may exert tumor-promoting effects (see below).

## 4. Expression of Sensory TRP Channels in Lung Cancer Cells

A functional expression of **TRPA1** in lung cancer cells has been detected in a broad panel of SCLC cell lines [55]. As mentioned above, SCLC cells show many neuroendocrine features, so that the expression of TRPA1 in these cells is in line with the well-established role of this channel in neurons [54,56,57,58,59] or neuroendocrine cells [60,61,62,63]. In SCLC cells, the activation of TRPA1 led to increased cell survival [55], in line with a potential role of TRPA1 in the regulation of apoptosis under stress conditions (see also next paragraph). Interestingly, in Lewis lung carcinoma cells both TRPA1 and **TRPM8** were functionally expressed and regulated critical cellular functions (metastasis, autophagy, energy metabolism) [64].

Expression of **TRPV1** has been demonstrated in lung adenocarcinoma cells [65,66,67]. TRPV1 expression has also been also demonstrated in lung fibroblasts [68] as well as in normal lung epithelium and sensory nerves (see above). Thus, the expression of this channel in lung tumors may be in part attributable to non-malignant stroma cells.

Moreover, Li et al. reported that **TRPV3** was overexpressed in NSCLC tissue as compared to adjacent noncancerous lung tissue [69] and that TRPV3 overexpressed was correlated with worse survival rates.

## 5. Activation of Sensory TRP Channels by Inhalative Carcinogens and Chemotherapeutics

TRPV1 and TRPA1 are involved in the detection of potentially harmful inhalants, e.g. by reactive electrophiles. However, these channels display a remarkable promiscuity with regard to their activators. For example, TRPA1 is activated by a large number of chemically unrelated substances (Table 1). Regarding the activation mechanism, the TRP channel can be stimulated by the direct reaction of the cysteine residues of the channel with an electrophilic agent [23,24,70], or by the reaction of the electrophilic compounds with constituents of the plasma membrane leading to the generation of secondary TRP activators [71,72].

**Figure 1:**
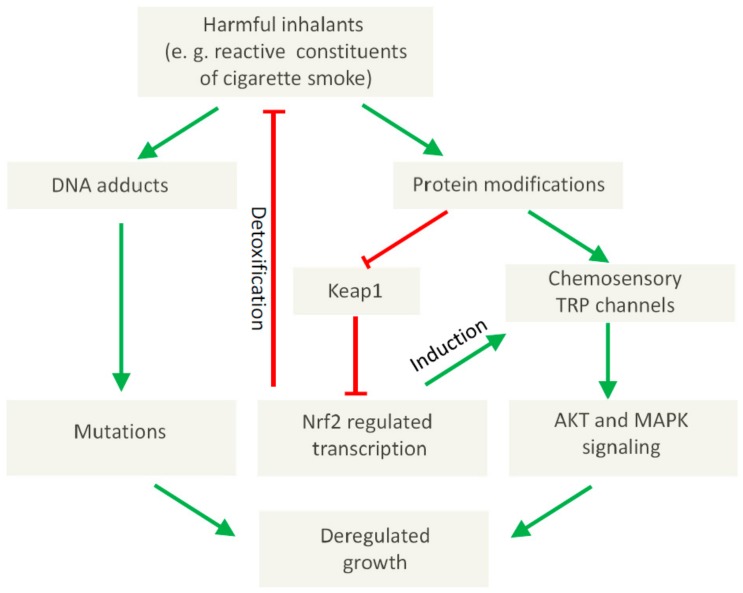
Proposed involvement of chemosensory TRP channels in tumor-promoting molecular effects elicited by harmful inhalants.

In the case of **TRPA1,** it has been shown that tumor-relevant compounds like cigarette smoke or DNA-damaging electrophiles can activate this channel [73,74,75]. Of note, the activation of TRPA1 in SCLC cells can promote cell survival [55] suggesting a potential role in tumor progression. Moreover, in lung adenocarcinoma cells, a direct interaction of TRPA1 with FGFR2 has been demonstrated, which may regulate the metastatic propensity of the cancer cells [82]. Thus, apart from the well-established direct genotoxic, tumor-initiating effects of DNA-damaging electrophiles, TRPA1 may also provide the mechanistic basis for a tumor-promoting role of these compounds via their potential to modify critical proteins like Keap1 or TRP channels (see Figure 1) with possible therapeutic implications. In this context, it is noteworthy that Takahashi et al. recently demonstrated an increased cellular resistance towards oxidative stress in breast and in lung cancer spheroids dependent on TRPA1 function [83]. In this latter paper, a self-amplifying mechanism was suggested, since reactive chemicals can activate NRF2-regulated transcription, which in turn leads to an induction of TRPA1 (Figure 1). Of note, the activation of NRF2 can also induce detoxifying enzymes [84,85] in this way inactivating potential carcinogens (Figure 1) leading to a complex picture, in which chemosensory TRP channels exert pivotal, regulatory functions.

## 6. Outlook

Chemosensory TRP channels such as TRPA1, TRPV1, and TRPV4 have emerged as important regulators of the epithelial integrity and mucociliary clearance of the airways. They are activated by exposure to potentially harmful inhalants (diesel exhaust, formalin, acrolein) or known lung carcinogens (electrophilic components of cigarette smoke). The activation of these channels is associated with inflammatory effects in the bronchial system and, more important in the context of lung tumorigenesis, some TRP members are involved in the regulation of oncogenic signaling pathways and may be involved in tumor-promoting effects.

Owing to the expression of TRP channels on the plasma membrane and the existence of more or less selective channel blockers or activators, these proteins are accessible to drug interventions. Even some approved drugs or novel drugs in clinical trials are available, which modulate some potentially tumor-relevant TRP channels, e.g., GRC 17536 (TRPA1) [86], XEN-D0501 (TRPV1) [80], SB705498 (TRPV1) [87]. Therefore, the elucidation of the cancer-relevant effects of TRP channels has a high translational impact and may define novel targets for therapeutic intervention.

## Figures and Tables

**Table 1:** Overview of some established TRPA1 activators with potential pathogeneic or therapeutic relevance for lung tumors.

Substance	Potential Source	References
Acrolein	Cigarette smoke	[73,74]
2-Chloroethyl-ethylsulfide	Analogue of sulfur mustard (chemical warfare agent)	[75]
Crotonaldehyde	Cigarette smoke	[73]
15-Deoxy-Delta(12,14)-prostaglandine J(2)	Endogenous inflammatory mediator	[76]
Formaldehyde	Cigarette smoke	[74,77]
Hydrogen peroxide	Endogenous inflammatory mediator	[76]
Nicotine	Cigarette smoke	[78,79]
Nitric oxide	Endogenous inflammatorymediator	[76]
Oxaliplatin	Chemotherapeutic agent	[80,81]

## Abbreviations

COPD: chronic obstructive pulmonary disease; NSCLC: non-small cell lung cancer; OS: overall survival; SCLC: small cell lung cancer; TRP: transient receptor potential channel.
